# Recipient *TIM4* signaling regulates ischemia reperfusion-induced ER stress and metabolic responses in liver transplantation: from mouse-to-human

**DOI:** 10.3389/frtra.2023.1176384

**Published:** 2023-05-19

**Authors:** Hirofumi Hirao, Shoichi Kageyama, Kojiro Nakamura, Kentaro Kadono, Hidenobu Kojima, Yao Siyuan, Douglas G. Farmer, Fady M. Kaldas, Kenneth J. Dery, Jerzy W. Kupiec-Weglinski

**Affiliations:** ^1^ Dumont-UCLA Transplantation Center, Department of Surgery, Division of Liver and Pancreas Transplantation, University of California, Los Angeles, Los Angeles, CA, United States; ^2^ Department of Surgery, Hepato-Biliary-Pancreatic Surgery and Transplantation, Kyoto University, Kyoto, Japan

**Keywords:** ischemia-reperfusion injury, liver transplantation, t-cell immunoglobulin and mucin domain containing 4, endoplasmic reticulum stress, orthotopic liver tranplantation

## Abstract

T-cell immunoglobulin and mucin (*Tim*)4 is expressed on APCs, including macrophages, as one of the main amplifiers in the mechanism of liver ischemia-reperfusion injury (IRI) following orthotopic liver transplantation (OLT). Though donor *Tim4* selectively expressed on Kupffer cells serves as a checkpoint regulator of innate immune-driven IRI cascades, its role on cells outside the OLT remains unclear. To dissect the role of donor vs. recipient-specific *Tim4* signaling in IR-induced stress and hepatocellular function, we employed a murine OLT model utilizing *Tim4*-knockout (KO) mice as either donor or recipient (WT → WT, WT → *Tim4*-KO, *Tim4*-KO → WT). In the experimental arm, disruption of donor *Tim4* attenuated IRI-OLT damage, while recipient *Tim4*-null mutation aggravated hepatic IRI concomitant with disturbed lipid metabolism, enhanced endoplasmic reticulum stress, and activated pro-apoptotic signaling in the grafts. In the *in vitro* study, murine hepatocytes co-cultured with *Tim4*-null adipose tissue showed enhanced C/EBP homologous protein (CHOP) expression pattern and susceptibility to hepatocellular death accompanied by activated caspase cascade in response to TNF-α stimulation. In the clinical arm, liver grafts from forty-one transplant patients with enhanced *TIM4* expression showed higher body mass index, augmented hepatic endoplasmic reticulum stress, enhanced pro-apoptotic markers, upregulated innate/adaptive immune responses, exacerbated hepatocellular damage, and inferior graft survival. In conclusion, although *TIM4* is considered a principal villain in peri-transplant early tissue injury, recipient *TIM4* signaling may serve as a savior of IR-triggered metabolic stress in mouse and human OLT recipients.

## 1. Introduction

The success of orthotopic liver transplantation (OLT) in patients with end-stage liver disease is often tempered by ischemia-reperfusion injury (IRI), an innate immune-driven hepatic sterile inflammation which contributes to primary graft dysfunction, higher incidence of rejection episodes, leading to shortages of life-saving donor organs (1). Although preventing liver IRI is essential for clinical OLT outcomes, the underlying mechanisms of IRI remain to be fully elucidated (2).

T-cell immunoglobulin and mucin domain-containing protein-4 (*Tim4*), identified initially as a *Tim1* ligand regulating T cell proliferation (3), is expressed on the surface of macrophages, DCs, B cells, and NK cells (4). Research studies show that *Tim4* is a key tissue-resident macrophage marker that binds to apoptotic cells via phosphatidylserine (5). Indeed, it plays an important role in a tightly regulated phagocytosis process, termed efferocytosis (6). In addition to its immune-regulatory role, the function of *Tim4* in maintaining lipid metabolism is attracting much attention. For instance, resident *Tim4 *+ macrophages in the adipose tissue (AT) are implicated in the regulation of lipogenesis, while hepatic TIM4+ macrophages are replaced by proinflammatory *Tim4*− macrophages in the progression of nonalcoholic fatty liver disease (7, 8). The function of “donor” *Tim4*, expressed primarily on Kupffer cells (KCs), has been investigated in liver injury models, including OLT (9, 10). Although recipient *Tim4* has been shown to be dispensable for allogenic murine heart graft survival (11), it remains unclear how recipient-derived *Tim4* signaling affects IR stress response in OLT recipients.

Here, we employed a mouse OLT model to investigate the distinctive role of donor vs. recipient *Tim4* signaling in IR-triggered OLT damage. Indeed, consistent with the aforementioned studies, *Tim4*-deficient donor livers implanted into WT recipients were relatively IRI-resistant. However, unexpectedly, WT livers transplanted into *Tim4*-null recipients showed augmented hepatic IRI, a disturbed lipid metabolic profile, enhanced endoplasmic reticulum (ER) stress responses, and curtailed survival. In a parallel clinical arm of forty-one OLT patients, donor livers with enhanced *TIM4* gene expression were profoundly IR-stress sensitive, resulting in exacerbated hepatocellular damage and significantly worse OLT outcomes. Thus, in this translational study, we have identified a novel regulatory function of recipient *TIM4* via metabolic and ER stress signaling in OLT recipients.

## 2. Materials and methods

### 2.1. Clinical liver transplant study

We retrospectively analyzed forty-one adult patients (≥18 years) who underwent OLT (May 2013–August 2015). As specified by UCLA protocols, all the recipients received routine standard of care and immunosuppressive therapy. Those who underwent re-transplantation were excluded from the study. Donor livers, procured from donation after a brain or cardiac death with standardized techniques, were perfused with and stored in UW solution (Niaspan; Bristol-Meyers Squibb Pharma, Princeton, NJ). Protocol Tru-Cut needle biopsies (Bx) from the left liver lobe were obtained pretransplant (after liver cold storage on the back table) and posttransplant (at about 2 h after portal reperfusion and before the abdominal closure). Liver Bx samples were analyzed by Western blots (WB) and qRT-PCR. Cold ischemia time was defined as the time from the perfusion of the donor liver with UW solution to its removal from the cold storage for implantation. Warm ischemia time was defined as the time from cold storage removal to the establishment of liver graft reperfusion. Recipient blood was collected before and after OLT, and sALT/sAST levels evaluated the hepatocellular function. Early allograft dysfunction (EAD) was defined by the presence of one or more of the following: bilirubin level of ≥10 mg/dl on POD (postoperative day) 7, Prothrombin time (PT)-International Normalized Ratio (INR) ≥ 1.6 on POD7, or AST/ALT level of >2,000 U/L within the first 7 days.

### 2.2. Animals

Wild-type (WT; Jackson Laboratory, Bar Harbor, ME) and *Tim4*-deficient male mice (*Tim4*-KO) (12), both at C57BL/6 background and 6–8 weeks of age, were used. Animals were housed in UCLA animal facility under specific pathogen-free conditions, and received humane care according to the criteria outlined in the “Guide for the Care and Use of Laboratory Animals” (NIH publication 86–23 revised 1985), while their use was reviewed and approved by UCLA Animal Research Committee.

### 2.3. Mouse orthotopic liver transplantation

We used a mouse model of ex-vivo hepatic cold storage followed by orthotopic liver transplantation (OLT), as described by our group (13). In brief, after the recipient liver was removed, the donor liver was placed orthotopically. The anastomosis of the suprahepatic vena cava was performed by running 10–0 nylon. The portal vein and the infrahepatic vena cava were both reconnected through a cuff technique. The bile duct reconstruction was completed with an intraluminal stent. The recipient anhepatic time was approximately 14 min. To mimic “marginal” human OLT setting, donor livers (WT or *Tim4*-KO) stored in UW solution (4°C/18 h) were transplanted to syngeneic recipient mice (WT or *Tim4*-KO). We used a syngeneic model to focus on putative homeostatic *Tim4* functions while avoiding confounding effects of host allo-immune MHC responses. Liver tissue and serum samples were collected at 6 h post-reperfusion, the peak of hepatocellular damage in this model (14) and at 24 h. Separate OLT recipient groups were monitored for survival. The sham group underwent the same procedures without OLT.

### 2.4. Hepatocellular function assay

Serum AST/ALT levels were measured with Infinity™ AST/ALT Liquid Stable Reagent (Thermo Scientific, Rockford, IL) and validated with Validate®GC3 (Maine Standards Company, LLC, ME).

### 2.5. OLT histology and IRI grading

Formalin-fixed paraffin-embedded liver sections (5 µm) were stained with hematoxylin and eosin (H&E). The severity of IRI was graded using Suzuki's criteria (15). Briefly, the degree of cytoplasmic vacuolization, sinusoidal congestion and parenchymal necrosis were scored from 0 to 4 (0: none, 1: minimal, 2: mild, 3: moderate, 4: severe).

### 2.6. TdT-mediated dUTP nick end labeling (TUNEL) assay

Cell death in liver sections (5 µm) was detected by In Situ Apoptosis Detection Kit (#MK500, TAKARA BIO USA) according to the manufacturer protocol. Results were scored semi-quantitatively by blindly counting the number of positive cells in 10 HPF (high-power field)/section.

### 2.7. Hepatocyte isolation and culture

Primary mouse hepatocytes were isolated using a two-stage collagenase perfusion method (14). Briefly, mouse liver was perfused with collagenase solution. After perfusion, liver was minced and dispersed in Geys balance salt solution. The cell suspension was centrifuged at 50*g* for 1 min to collect hepatocytes. The hepatocytes were cultured on type 1 collagen coated plate. In some experiments, hepatocytes were stimulated with TNF-α (#575204, Biolegend, San Diego, CA) for 6 h.

### 2.8. Adipose tissue culture

Adipose tissue (AT) conditioned media was prepared, as reported (16). Briefly, epididymal fat pads collected from WT or *Tim4*-KO mice were cut into 2–3 mm^3^ pieces. For relaxation, those fragments were incubated in Dulbecco's Modified Eagle Medium (DMEM) for 24 h, then washed with PBS and re-incubated in DMEM for 24 h. After re-incubation, culture media was filter-sterilized and collected as adipose tissue conditioned media (AT-CM) for hepatocyte culture.

### 2.9. Quantitative Rt-PCR analysis

RNA extracted with NucleoSpin® RNA (#740955.50, TAKARA BIO USA, Inc, Mountain View, CA) was reverse-transcribed into cDNA with the PrimeScript RT Reagent Kit (#RR037, TAKARA BIO). Quantitative PCR was performed using QuantStudio 3 (Applied Biosystems, Foster City, CA). The primer sequences are listed in Supplementary Table S1. The expression of the target gene was normalized to the housekeeping *Hprt* (mouse) or *GAPDH* (human).

### 2.10. Western blot assay

Proteins were extracted from tissue/cell samples, and their concentration was measured using BCA Protein Assay Kit (#23227, Thermo Fisher Scientific, Waltham, MA). An equal amount of protein was electrophoresed, blotted, incubated with primary Ab, and secondary HRP-conjugated Ab, and developed. Primary Abs used are listed in Supplementary Table S2. To compare target protein expression in multiple human OLT samples, densitometry quantification was conducted, as reported (14, 17).

### 2.11. ELISA

Serum concentration of IL-10 was measured by ELISA kit (#431414, Biolegend, San Diego, CA) according to the manufacturer protocol.

### 2.12. Measurement of serum-free fatty acids

Serum free fatty acid levels were measured by Free Fatty Acid Fluorometric Assay Kit (#700310, Cayman Chemical, Ann Arbor, MI) according to the manufacturer protocol.

### 2.13. Immunohistochemistry

Paraffin-embedded mouse liver sections were stained with rabbit anti-CD36 Ab, mouse anti-CHOP Ab or mouse anti-PPARγ antibodies with M.O.M.® (Mouse on Mouse) ImmPRESS® HRP (Peroxidase) Polymer Kit (MP-2400, Vector Laboratories, Inc. Newark, CA) according to the manufacturer's protocol. The signal was visualized by diaminobenzidine tetrahydrochloride (DAB, D5905, MilliporeSigma, Burlington, MA), and the nucleus was counterstained with hematoxylin.

### 2.14. Immunocytochemistry

Hepatocytes isolated from WT mice were seeded into the chamber slide (#177445, Thermo Fisher Scientific, Waltham, MA) and fixed with 4% paraformaldehyde for 10 min. Cells were stained with primary Abs, and Alexa-conjugated secondary Abs were used to visualize the signal. The antibodies used in the study are listed in Supplementary Table S2.

### 2.15. Statistical analysis

For mouse experiments, the comparison between the two groups were assessed using the Mann-Whitney *U* test or 1 way-ANOVA followed by Tukey's HSD test. For human data, continuous values were analyzed by the Mann-Whitney *U* test or 2 way-ANOVA followed by Bonferroni *post hoc* test and categorical variables by Fisher's exact test. Cumulative survival rates were estimated using the Kaplan-Meier method, and survival curves were analyzed using log-rank tests. All *P* values were 2-tailed, and *P* less than 0.05 was considered statistically significant.

### 2.16. Study approval

All human studies were approved by the UCLA Institutional Research Board (IRB protocol 13-000143), and written informed consent was received from participants before inclusion in the study. All mouse experiments were approved by the UCLA Animal Research Committee (ARC #1999-094).

## 3. Results

### 3.1. Disruption of donor *Tim4* signaling attenuates Ir-triggered hepatocellular damage in murine OLT

**Figure 1:**
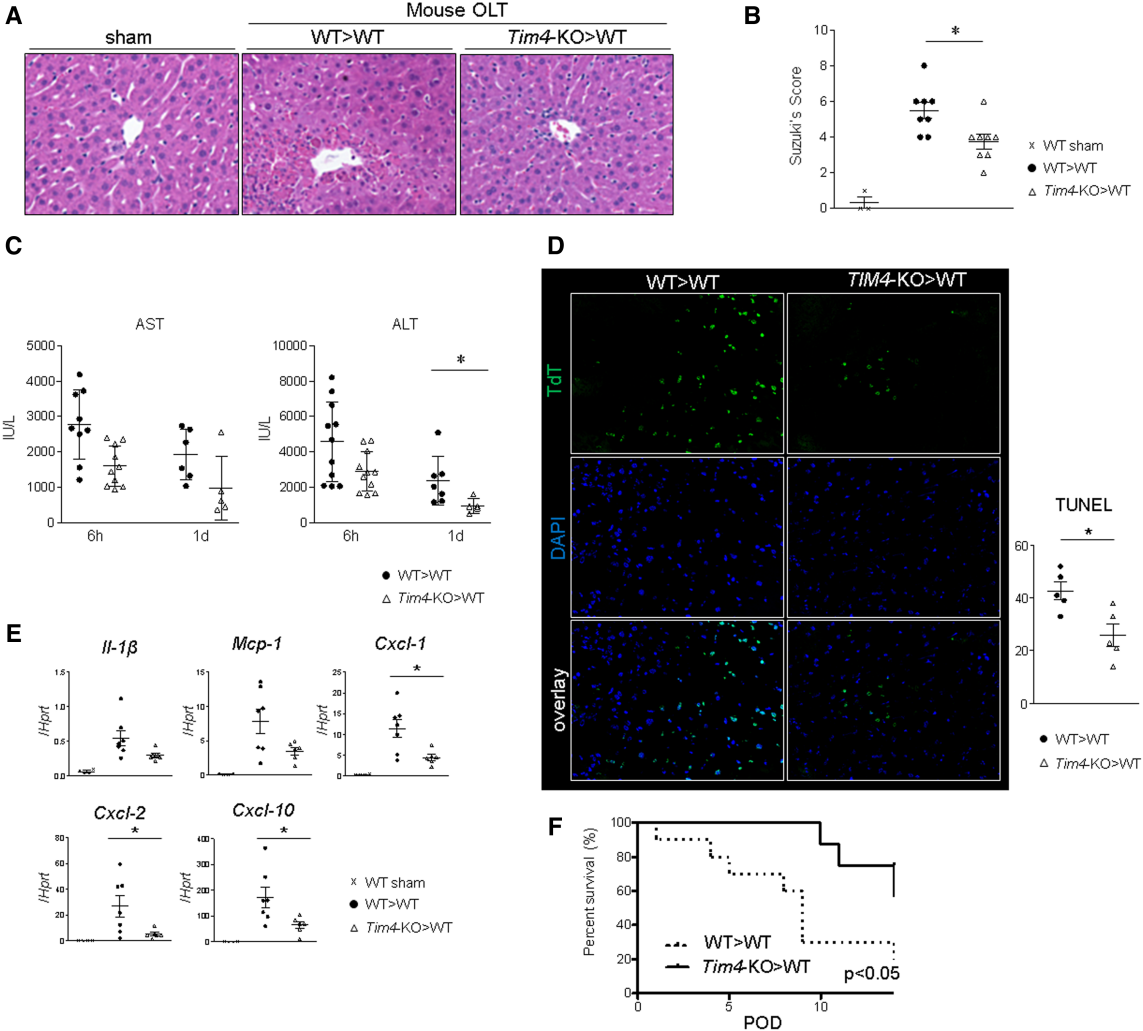
Donor *Tim4*-null mutation alleviates the hepatocellular damage and inflammatory response in IR-stressed mouse OLT: WT or *Tim4*-KO donor livers, stored in UW solution (4°C/18 h), were transplanted into WT recipients. OLT samples were collected at 6 h and 24 h post-reperfusion. (**A**) sAST and sALT levels (IU/L). (**B**) Representative H&E staining (original magnification, ×100) of sham livers and OLT (WT > WT, *Tim4*-KO > WT). (**C**) Suzuki's histological grading of liver IRI (*n* = 8/group). Data shown as mean ± SEM (*p < 0.05, 1-way ANOVA followed by Tukey's HSD test). (**D**) Representative TUNEL images and quantification of TUNEL + cells (*n* = 5/group). Data shown as mean ± SEM (*p < 0.05, Mann-Whitney U test). (**E**) qRT-PCR-assisted detection of mRNA coding for *Il-1β, Mcp1, Cxcl1, Cxcl2 and Cxcl10*. Data were normalized to *Hprt* gene expression. Sham: *n* = 4/group; OLT: *n* = 6–7/group. Data shown as mean ± SEM (*p < 0.05, Mann-Whitney U test). (**F**) Recipient mice after OLT were monitored for 14 days and cumulative survival was analyzed (Kaplan-Meier method). Dotted line: WT > WT; solid line *Tim4*-KO > WT (*n* = 10/group, *p < 0.05, log-rank test).

Livers from *Tim4*-deficient mice (*Tim4*-KO; C57BL6), stored for 18 h at 4C, were transplanted into groups of syngeneic WT recipients, followed by sampling at 6 h and 1 day after OLT. Histological analyses showed hepatic *Tim4* deficiency decreased sinusoidal congestion, edema, vacuolization, and hepatocellular necrosis in IR-stressed OLT (Figure 1A). These findings translated to improved Suzuki's histological score of IR-damage (Figure 1B), attenuated sAST and sALT release (Figure 1C), suppressed frequency of TUNEL + cells (Figure 1D), depressed hepatic mRNA levels coding for *Il-1β*, *Mcp1*, *Cxcl1*, *Cxcl2* and *Cxcl10* (Figure 1E), and prolonged OLT survival (Figure 1F). These results confirm *Tim4* expressed primarily by hepatic KCs functions as a proinflammatory sentinel in the mechanism of IR-triggered OLT damage.

### 3.2. Recipient *Tim4* deficiency aggravates the hepatocellular damage in murine OLT

**Figure 2:**
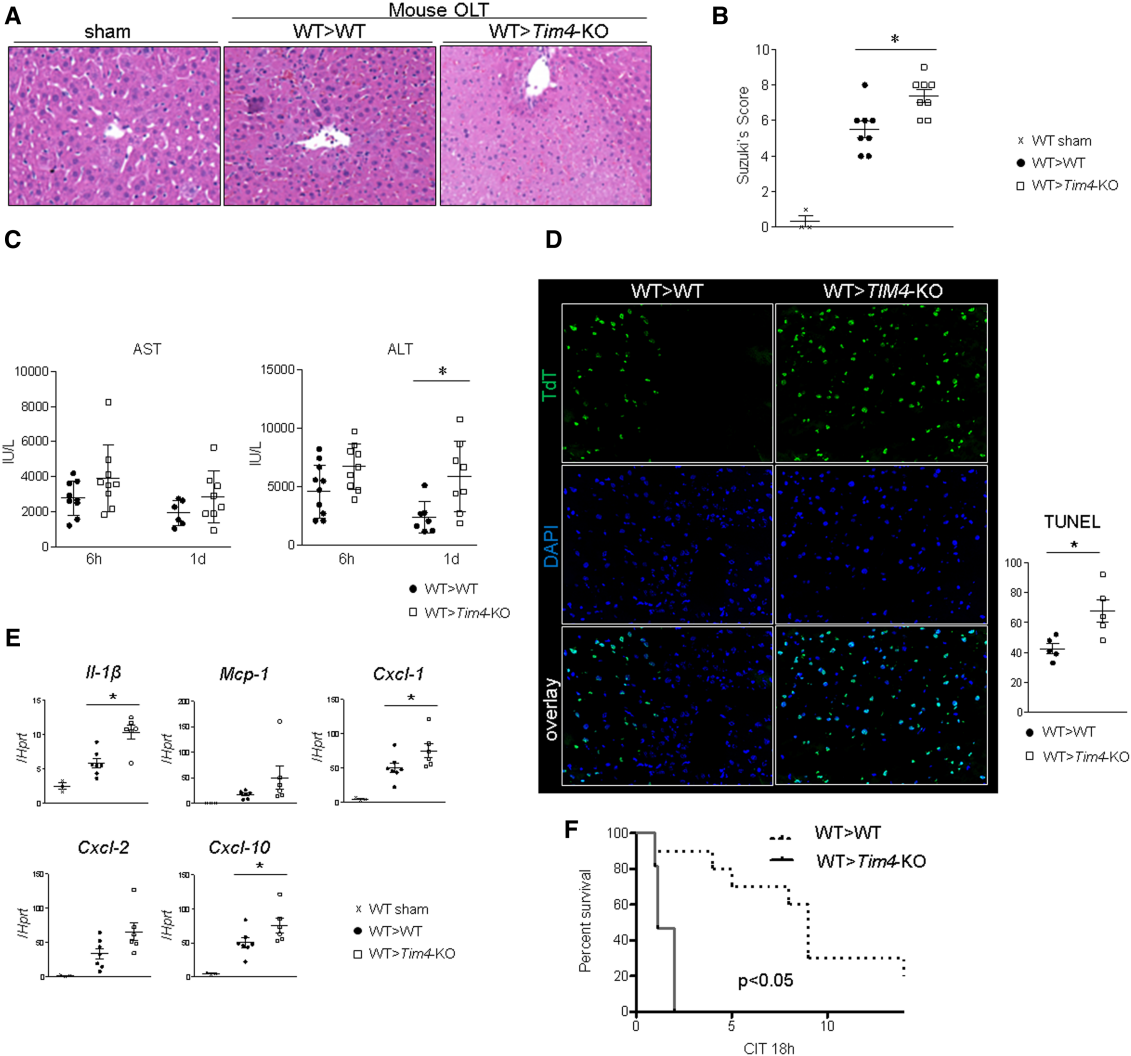
Recipient *Tim4* disruption aggravates the hepatocellular damage and inflammatory response in IR-stressed mouse OLT: (**A**) sAST and sALT levels (IU/L). (**B**) Representative H&E staining (original magnification, ×100) of sham livers and OLT (WT > WT, *Tim4*-KO > WT). **(C)** Suzuki's histological grading of liver IRI (*n* = 8/group). Data shown as mean ± SEM (*p < 0.05, 1-way ANOVA followed by Tukey's HSD test). **(D)** Representative TUNEL images and quantification of TUNEL + cells (*n* = 5/group). Data shown as mean ± SEM (*p < 0.05, Mann–Whitney *U*-test). **(E)** qRT-PCR-assisted detection of mRNA coding for *Il-1β, Mcp1, Cxcl1, Cxcl2* and *Cxcl10*. Data were normalized to *Hprt* gene expression. Sham: *n* = 3/group; OLT: *n* = 6–7/group. Data shown as mean ± SEM (*p < 0.05, Mann–Whitney *U*-test). (**F**) Recipient mice were monitored for 14 days and cumulative survival was analyzed (Kaplan–Meier method). Dotted line: WT > WT; solid line WT > *Tim4*-KO (*n* = 10/group, *p < 0.05, log-rank test).

To focus on the role of recipient-derived *Tim4* signaling, IR-stressed WT donor livers were implanted into groups of *Tim4*-deficient or *Tim4*-proficient (WT) mice. Unexpectedly, recipient *Tim4* deficiency markedly deteriorated OLT function and outcomes, evidenced by increased sinusoidal congestion, edema, vacuolization, and hepatocellular necrosis (Figure 2A), higher Suzuki's histological score of hepatic IRI (Figure 2B), augmented sAST/sALT release (Figure 2C), increased frequency of TUNEL + cells (Figure 2D) and enhanced hepatic mRNA levels coding for *Il-1β*, *Mcp1*, *Cxcl1*, *Cxcl2* and *Cxcl10* (Figure 2E). Strikingly, *Tim4*-KO recipients died within 2 days post-OLT, while all WT recipients (WT > WT) showed considerably longer survival (Figure 2F). The detailed mouse OLT data points are shown (Supplementary Table S3).

### 3.3. Disruption of recipient *Tim4* signaling disturbs lipid metabolism in murine OLT

**Figure 3:**
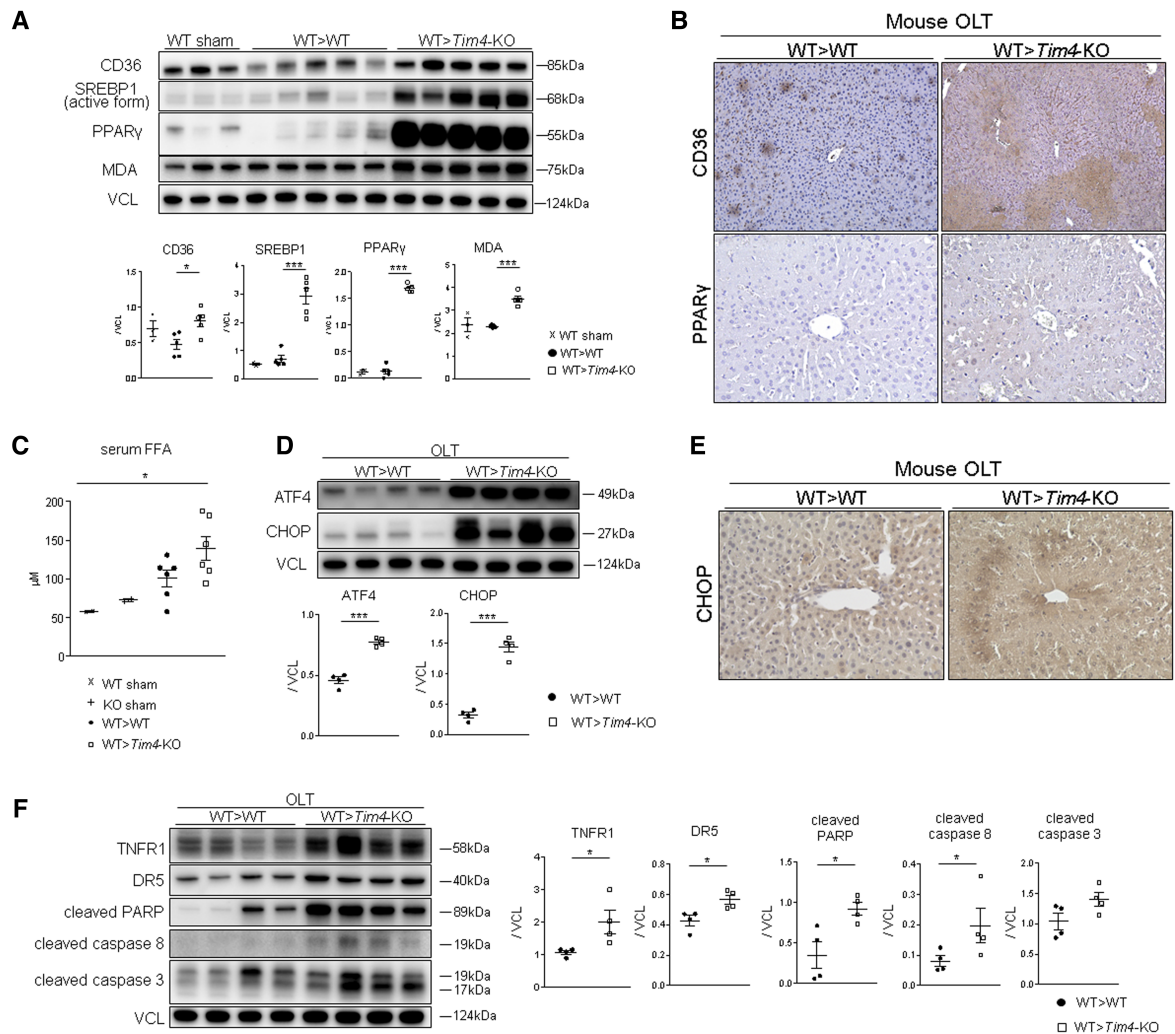
Recipient *Tim4*-null mutation provokes hepatic lipid metabolism pathway and augments ER stress and apoptotic markers in OLT: (**A**) Western blot of CD36, SREBP1, PPARγ and MDA in sham and OLT livers (WT > WT, WT > *Tim4*-KO). The relative intensity to VCL was calculated. Data shown as mean ± SEM (*p < 0.05, ***p < 0.001, 1-way ANOVA followed by Tukey's HSD test). (**B**) Representative immunohistochemistry of CD36 and PPAR-γ in OLT livers (*n* = 2/group). (**C**) Serum FFA levels in sham and OLT mouse recipients. Data shown as mean ± SEM (*p < 0.05, 1-way ANOVA followed by Tukey's HSD test). (**D**) WB-assisted detection of ATF4, CHOP and VCL. The relative intensity to VCL was calculated. Data shown as mean ± SEM (***p < 0.001, Mann–Whitney *U*-test). (**E**) Representative staining for CHOP in OLT (*n* = 3/group). (**F**) WB of TNFR1, DR5, cleaved PARP, caspase 8, caspase 3 and VCL in OLT. The relative intensity to VCL was calculated. Data shown as mean ± SEM (*p < 0.05, Mann–Whitney *U*-test).

Having found opposite phenotypes in IR-stressed mouse OLT and based on previous reports (4), we hypothesized that disruption of recipient *Tim4* signaling disturbs lipid metabolic pathways that normally suppress the proinflammatory response to IR stress and attenuates OLT damage (18). Indeed, OLT expression of CD36 (also known as fatty acid translocase; FAT), a key mediator of cell membrane free fatty acid (FFA) and oxidized low-density lipoprotein (ox-LDL) synthesis was significantly upregulated in *Tim4*-null recipients (Figure 3A). Other remarkable differences occurred for sterol regulatory element-binding protein 1 (SREBP1) and peroxisome proliferator-activated receptor gamma (PPARγ), both of which are involved in lipogenesis (Figures 3A,B). Importantly, serum FFA levels after OLT were significantly higher in *Tim4*-KO as compared with WT counterparts (Figure 3C), while recipient *Tim4*-null mutation enhanced hepatic lipid peroxidation, evidenced by increased malondialdehyde (MDA) levels (Figure 3A). These findings suggest that recipient *Tim4* signaling is indispensable for maintaining metabolic homeostasis to prevent excessive lipid accumulation in IR-stressed OLT.

### 3.4. Recipient *Tim4* deficiency augments hepatic ER stress and provokes apoptotic response *in vivo*

Since previous reports document that SREBP1 and ER stress coordinately enhance lipotoxicity (19), we confirmed that both ATF4 and CHOP ER stress markers were significantly upregulated in *Tim4*-KO mice (Figure 3D). Immunohistochemistry of IR-stressed OLT revealed hepatocytes as the primary source of CHOP expression (Figure 3E). In addition, *Tim4*-null mice exhibited augmented cell death receptors (e.g., TNFR1 or DR5) and pro-apoptotic molecules, such as active PARP [Poly (ADP-ribose) polymerase], caspase 8 and caspase 3 (Figure 3F).

### 3.5. *Tim4* deficiency in adipose tissue enhances hepatocyte SREBP1/CHOP and promotes TNF-α mediated cell death *in vitro*

**Figure 4:**
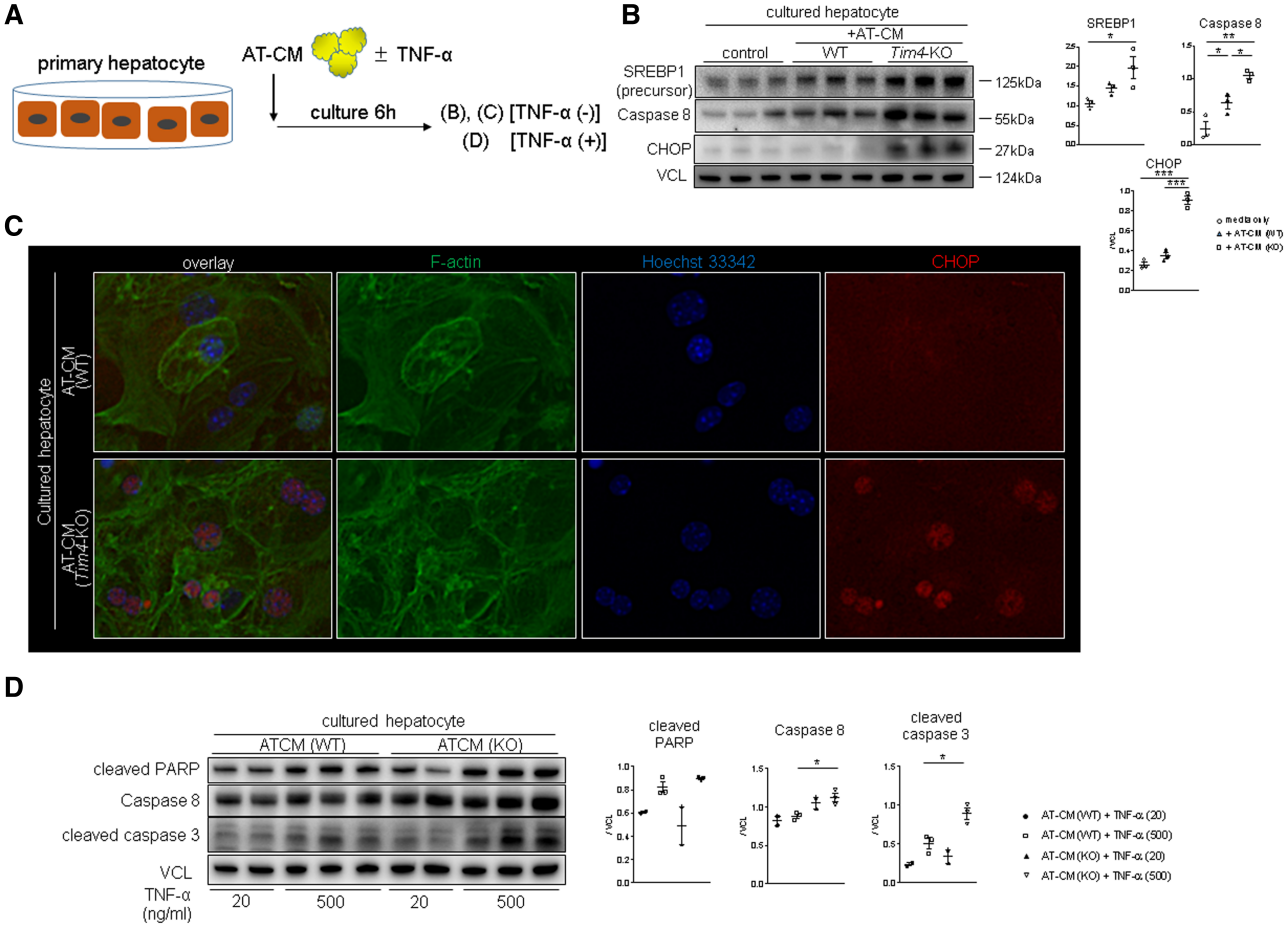
*Tim4* deficient adipose tissue enhances hepatocyte SREBP1/CHOP and promotes TNF-α mediated cell death *in vitro*: (**A**) the scheme of the vitro experiment. (**B**) WB-assisted detection of SREBP1, caspase 8, CHOP and VCL. The relative intensity to VCL was calculated. Data shown as mean ± SEM (*p < 0.05, **p < 0.01, ***p < 0.001, 1-way ANOVA followed by Tukey's HSD test). (**C**) Representative immunocytochemistry of F-actin (green), nucleus (blue) and CHOP (red) in murine hepatocyte cultured with AT-CM from WT or *Tim4*-KO mice (*n* = 2). Original magnification, ×400. (**D**) WB of cleaved PARP, caspase 8, cleaved caspase 3 and VCL in cultured hepatocytes. The relative intensity to VCL was calculated. Data shown as mean ± SEM (*p < 0.05, 1-way ANOVA followed by Tukey's HSD test).

To further elucidate the underlying mechanism of *Tim4*-dependent regulation of lipid metabolism, we co-cultured WT mouse hepatocytes with adipose tissue-conditioned media (AT-CM) from WT vs. *Tim4*-deficient mouse donors (Figure 4A). As shown in Figure 4B, hepatocytes co-cultured with AT-CM from *Tim4*-null mice showed enhanced SREBP1, caspase 8, and CHOP expression compared to AT-CM from WT counterparts. Figure 4C illustrates representative immunocytochemistry of CHOP in cultured hepatocytes after AT-CM co-culture. Next, we asked whether AT-CM might confer the susceptibility to hepatocellular death. Indeed, as shown in Figure 4D, hepatocytes primed with AT-CM from *Tim4*-KO mice were more sensitive to TNF-α mediated hepatocellular death than from WT counterparts. This was evidenced by increased cleaved PARP, caspase 8, and cleaved caspase 3 expression. This data illustrates AT-expressing *Tim4* regulates the hepatocellular metabolic program, ER stress, and apoptotic pathway.

### 3.6. *TIM4* levels correlate with the hepatocellular function in human OLT recipients

**Figure 5:**
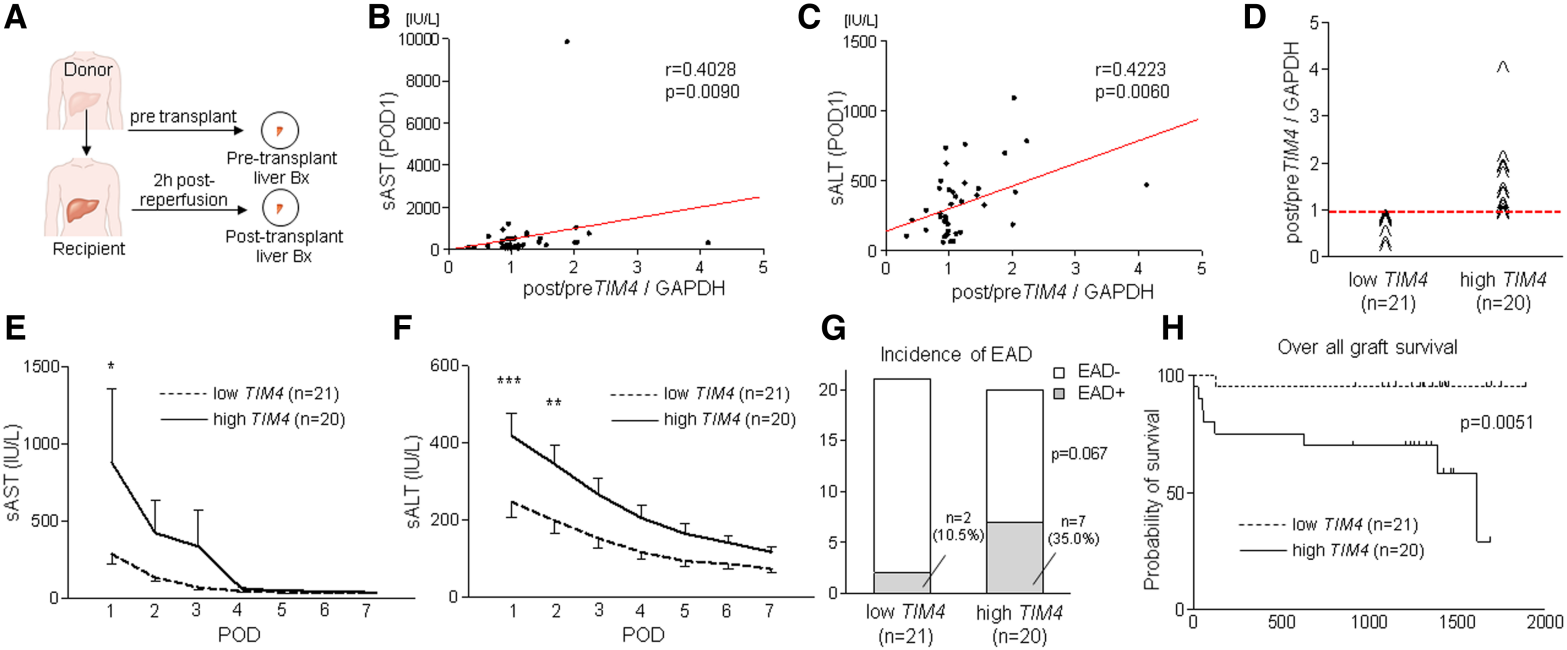
Peri-transplant *TIM4* gene levels are associated with the hepatocellular function and OLT outcomes: (**A**) Pre-transplant (post–cold storage) and post-transplant (2 h after reperfusion) liver biopsies (Bx) were collected from forty-one OLT patients and analyzed for *TIM4* by qRT-PCR with normalization to *GAPDH*. The relationship between *TIM4* and sAST (**B**) and sALT (**C**) at POD1. *r*: Spearman correlation coefficient. (**D**) Human OLT Bx (2 h post-reperfusion) were classified into low (*n* = 21) and high (*n* = 20) *TIM4* expression groups. (**E,F**) Serum AST and ALT levels at POD1-7 (*p < 0.05, **p < 0.01, ***p < 0.001, 2-way ANOVA, Bonferroni *post hoc* test; data shown as mean ± SEM. (**G**) Incidence of early allograft dysfunction (EAD) (Fisher's exact test). (**H**) The cumulative probability of overall graft survival. The solid line indicates *TIM4*-high, while the dotted line depicts *TIM4*-low OLT patient cohorts (Kaplan–Meier method, log-rank test).

Having demonstrated the functional role of *Tim4* expression in mouse IRI-OLT, we next aimed to validate its relevance by screening retrospectively forty-one human OLT cases. Liver Bx samples were assessed for *TIM4* with *GAPDH* normalization by qRT-PCR (Figure 5A). Post-transplant (2 h after reperfusion) expression levels were compared to pre-transplant (after cold storage) samples (Post/Pre) to determine the perioperative *TIM4* profile. Consistent with the mouse data (Supplementary Figure S1), peritransplant hepatic *TIM4* levels correlated positively with early OLT function, assessed by sAST (*r *= 0.4028, *p* = 0.0090, Figure 5B) and sALT (*r* = 0.4223, *p* = 0.0060, Figure 5C) at postoperative day 1 (POD1). Then, based on the *TIM4* gene expression pattern, liver Bx samples were divided into *TIM4*-low (*n* = 21) and *TIM4*-high (*n* = 20) groups, according to the median split (Figure 5D). Patients' demographic data and clinical parameters are shown in Supplementary Tables S4, S5. We found no correlation between *TIM4* levels and recipient surgical parameters, including age, gender, race, ABO compatibility, pre-transplant blood tests, preoperative hospital stay, cold ischemia time, warm ischemia time, or blood transfusions during the surgery. However, BMI was significantly higher in the *TIM4*-high group (*p* = 0.020) (Supplementary Table S4). There was no correlation between *TIM4* grouping and other donor data (Supplementary Table S5), including age, gender, race, BMI, pre-procurement blood tests, or donation status (after circulatory or brain death). Of note, the *TIM4*-high expression clinical cohort had significantly higher sAST at POD1 and sALT levels at POD1-2 (Figures 5E,F), reflecting a deteriorated OLT function. Although the difference failed to reach statistical significance, *TIM4*-high cases experienced an increased frequency of EAD compared to the *TIM4*-low group (Figure 5G, 10.5% vs. 35.0%, *p* = 0.0670). We analyzed graft survival rates to examine the relationship between *TIM4* expression and OLT outcomes (median follow-up, 1,269 days; range, 3–1,892 days). Strikingly, the *TIM4*-high patient cohort experienced significantly inferior overall graft survival (Figure 5H, *p* = 0.0051). None of the patients underwent secondary OLT.

### 3.7. *TIM4* regulates innate—adaptive immune interphase in human OLT recipients

**Figure 6:**
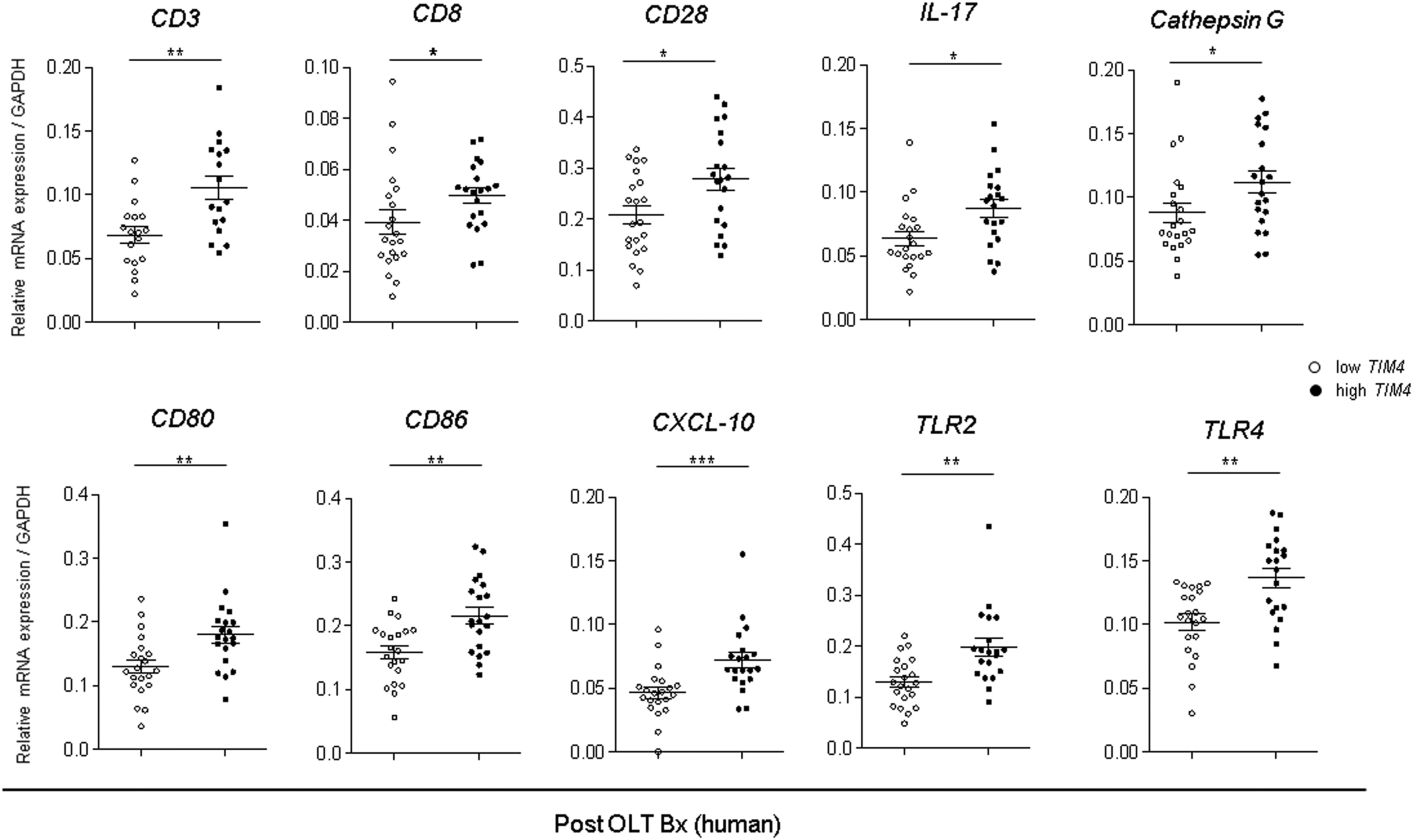
Augmented innate and adaptive gene activation in human OLT is accompanied by high *TIM4* levels: qRT-PCR-assisted detection of mRNA coding *for CD3, CD8, CD28, IL-17, Cathepsin G, CD80, CD86, CXCL-10, TLR2*, and *TLR4*. Data normalized to *GAPDH* gene expression are shown in dot plots and bars indicative mean ± SEM (*p < 0.05, ** *p* < 0.01, *** *p* < 0.001, Mann–Whitney *U*-test).

We also analyzed the innate, and adaptive immune gene expression profiles in human liver Bx obtained during OLT (Figure 6). Consistently, *TIM4*-high grafts exhibited increased mRNA levels coding for T cell activation markers, *CD3* (*p* = 0.0246), *CD8* (*p* = 0.0628), *CD28* (*p* = 0.0216), and *IL-17* (*p* = 0.0216), macrophage activation markers, *CD80* (*p* = 0.0188), *CD86* (*p* = 0.0072), *CXCL10* (*p* = 0.0257), *TLR2* (*p* = 0.0018) and *TLR4* (*p* = 0.0024), as well as the neutrophil activation marker, *Cathepsin G* (*p* = 0.0196). Hence, the *TIM4*-high phenotype was accompanied by accelerated post-reperfusion innate/adaptive immune activation and enhanced hepatocellular damage in the early post-OLT phase.

### 3.8. *TIM4* expression is associated with CHOP, MDA and pro-apoptotic markers in human OLT

**Figure 7:**
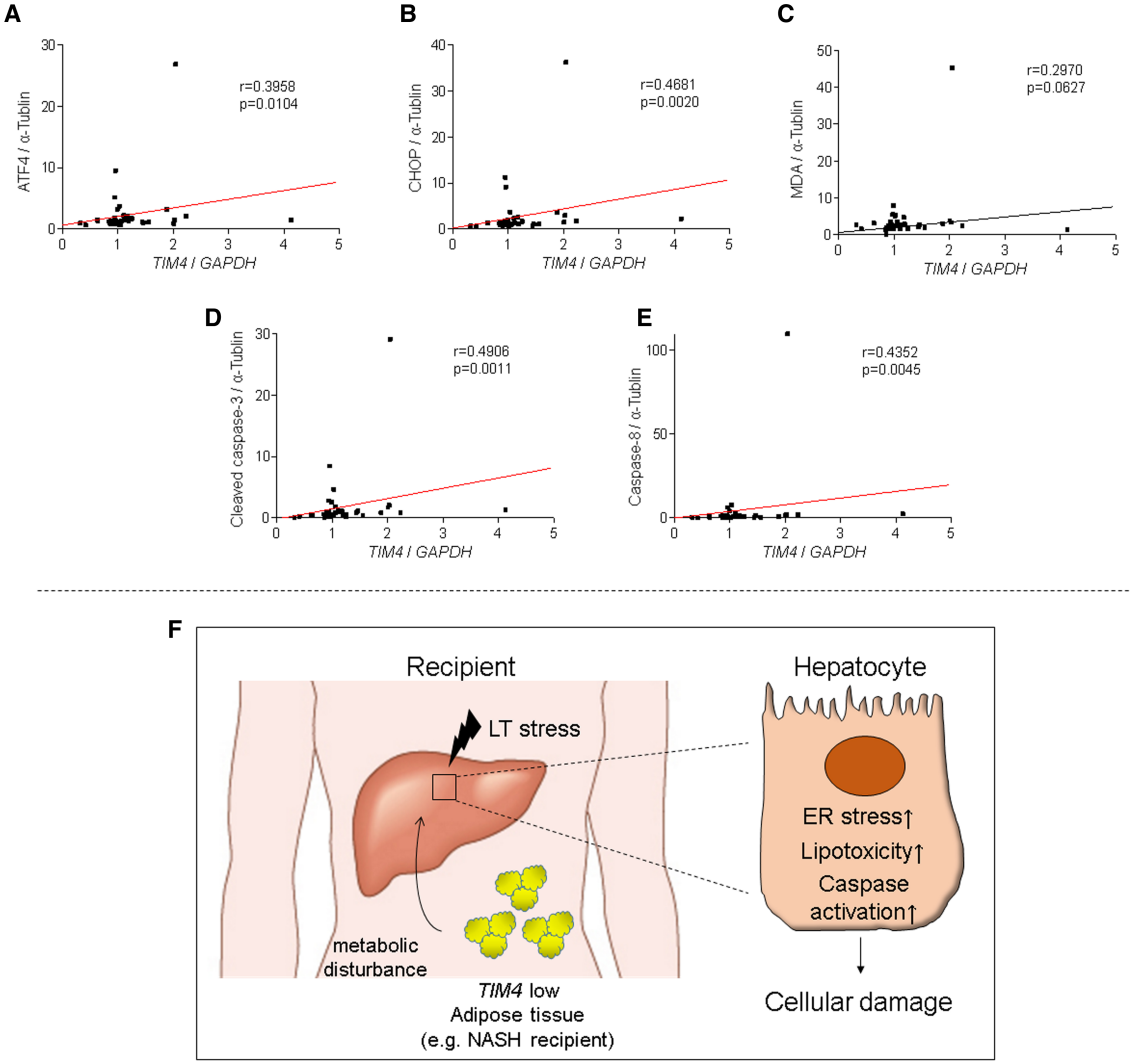
*TIM4* expression is positively correlates with hepatic ATF4, CHOP, MDA, caspase 8 and cleaved caspase 3 levels in human OLT: the relationship between peri-transplant *TIM4* levels and ATF4 (**A**) CHOP (**B**) MDA (**C**) cleaved caspase 3 (**D**) and caspase 8 (**E**) expression in OLT at 2 h after reperfusion. *r*: Spearman correlation coefficient. (**F**) A simplified scheme of how *TIM4* signaling may affect IR-stress and hepatocellular damage in OLT recipients.

Since we showed the relationship between *TIM4* and ATF4, CHOP, cleaved caspase 3, caspase 8, and MDA expression in the experimental murine settings, we then analyzed our clinical liver Bx samples to validate the aforementioned findings. Consistent with the mouse data, peritransplant *TIM4* levels at 2 h after reperfusion in human OLT correlated positively with hepatic ATF4 (*r* = 0.3958, *p* = 0.0104), CHOP (*r* = 0.4681, *p* = 0.0020), MDA (*r* = 0.2970, *p* = 0.0627), cleaved caspase 3 (*r* = 0.4906, *p* = 0.0011) and caspase 8 (*r* = 0.4352, *p* = 0.0045) (Figures 7A–E).

## 4. Discussion

This translational study uncovered a distinctive function of recipient-derived TIM4 signaling in IR-stressed in murine and clinical OLT settings. In the experimental arm in mouse OLT recipients, donor *Tim4* disruption attenuated IRI (Figure 1), whereas recipient *Tim4*-null mutation deteriorated the hepatocellular function and augmented innate immune activation, evidenced by liver histology, frequency of TUNEL + cells, and a proinflammatory gene-phenotype. Furthermore, enhanced OLT damage was accompanied by increased intrahepatic *Tim4* expression, ER stress, and disturbed hepatic lipid metabolic pathway in *Tim4*-KO hosts. In the clinical arm of forty-one liver transplant patients, increased hepatic *TIM4* expression correlated with higher BMI, worsened OLT function, and enhanced innate/adaptive immune activation while trending towards higher frequency of EAD, and inferior overall graft survival, as compared with the *TIM4*-low patient cohort (Figures 5–7).

Several lines of evidence have implicated *Tim4* signaling as a proinflammatory sentinel. First, compared with *Tim4*-proficient macrophages, *Tim4*-deficient peritoneal macrophages released lower proinflammatory cytokines in response to LPS stimulation *in vitro* (5). Next, inhibition of *Tim4* signaling on DCs enhanced Treg induction and contributed to prolonged skin allograft survival (20), whereas *Tim4*+ B cells released more IFN-γ and shifted CD4+ T cells towards less IL-4, IL-10 and FoxP3 levels (21). Moreover, Zhang et al. have demonstrated *Tim4* was indispensable for NKT cell function (22). However, our study quite unexpectedly found that IR-stressed WT donor livers showed augmented hepatocellular damage and poor survival rates when implanted into *Tim4*-deficient mouse recipients (despite utilizing a syngeneic OLT model). *Tim4* is essential for phagocytosis and subsequent IL-10 release by the engulfment of apoptotic cells (23). As IL-10 serves as an anti-inflammatory sentinel in the pathogenesis of liver IRI (24, 25), we hypothesized that increased liver damage seen in *Tim4*-null hosts may have been due to impaired phagocytosis and depressed IL-10 release. However, IL-10 sera levels in OLT recipient groups (WT > WT or WT > *Tim4*-KO) were comparable (Supplementary Figure S2).

Recent studies have shed light on the unique role of macrophage *Tim4* as a major regulator of lipid homeostasis (26, 27). Magalhaes et al. demonstrated that *Tim4* expression on AT macrophages, but not KC or peritoneal macrophages, was indispensable for regulating post-prandial cholesterol levels (26). Hence, we postulated that metabolic disturbances due to the absence of *Tim4* in recipients' AT might have worsened OLT outcomes. Indeed, we found severe lipid dysregulation in WT livers transplanted into *Tim4*-null mouse recipients, evidenced by augmented hepatic CD36, SREBP1, PPARγ, and MDA expression. The CD36, a transport protein for FFA closely involved in the development of nonalcoholic steatohepatitis (NASH) (28), and serum FFA were significantly upregulated in *Tim4*-null mice. Although the expression of hepatic PPARγ is relatively low in steady-state conditions, PPARγ is known to be induced by high-fat diet (HFD, Supplementary Table S6) or oleic acid treatment to promote lipid utilization (29). In our OLT model, PPARγ and MDA markers for lipid peroxidation (30) were profoundly enhanced in *Tim4*-null hosts. Taken together, *Tim4*-deficient OLT recipients were suffering from IR stress-induced excessive dyslipidemia. Although Thornley et al. reported that *Tim4*-KO and *Tim4* proficient (WT) hosts showed comparable cardiac allograft survival (11), liver grafts controlling multiple metabolic pathways may be particularly sensitive to IR-triggered metabolic disorders.

The question arises of how lipid metabolism-related molecules could affect the hepatocellular damage. We found ER stress markers were highly upregulated in *Tim4*-null recipients. With ER stress playing a central role in lipid and protein biosynthesis (31), its capability to newly synthesize lipids or proteins decreases in response to cellular stress, accumulating unfolded proteins in ER lumen. With ER stress implicated in various pathologies, including hepatic IRI (17, 32), the expression of CHOP, one of ER stress markers, plays a central role in apoptosis (33). Indeed, CHOP-deficient mice were resistant to warm IRI concomitant with suppressed apoptosis (34). Meanwhile, SREBP1 is anchored to ER membrane as an inactive precursor protein and cleaved into transcriptionally active SREBP1 by SREBP cleavage-activating protein (35, 36) to amplify ER stress and enhance cellular lipotoxicity (19, 37). In line with these reports, disruption of recipient *Tim4* signaling augmented ER stress and SREBP1, accompanied by activated pro-apoptotic proteins (Figure 3). These vivo findings were reproducible in our co-culture system where AT-CM adjunct from *Tim4*-deficient mice augmented hepatocyte SREBP1/CHOP expression and conferred susceptibility to hepatocellular death (Figure 4). However, the underlying mechanism that bridges *Tim4* signaling from AT to hepatocytes remains to be elucidated since FFA levels between AT-CM from WT and *Tim4*-KO mice were comparable (Hirao et al. unpublished). Recently, the novel role of AT in regulating microRNAs (miRNAs) has been identified, where AT-derived miRNAs could regulate gene expression in distal organs (38, 39). Future studies should address putative differences in miRNA profiles between WT and *Tim4*-null AT.

In OLT patients, *TIM4* levels were associated with clinical outcomes (Figure 5), while *TIM4*-high phenotype was accompanied by augmented hepatic ER stress, lipid peroxidation, and pro-apoptotic markers (Figure 7F). Interestingly, BMI was significantly elevated in the *TIM4*-high clinical cohort. These results are in agreement with our experimental data where liver grafts in HFD-fed mouse recipients exhibited increased hepatocellular necrosis, augmented hepatic *TIM4*, SREBP1, PPARγ and CHOP expression, as well as much curtailed survival as compared to liver grafts in ND-fed counterparts (Supplementary Figure S3). These data suggest that obesity (e.g., in NASH) might have a common pathology with HFD and *Tim4*-deficiency. Since the number of NASH cases was small in our clinical study, future detailed assessment in a larger cohort is warranted. The limitation of our study is that unlike TIM4 expressed primarily on donor liver KCs, it was difficult to define the source of *Tim4* “recipient” cells, while AT may have reflected a mixture of *Tim4*-expressing cells. Nevertheless, our findings imply that NASH, a leading indicator for future liver transplantation worldwide (40, 41), is prone to display a *TIM4*-low phenotype highly sensitive to IRI-OLT metabolic stress. Hence, targeting donor specific *TIM4* rather than global *TIM4* may provide the ultimate protection against IR stress and OLT damage.

In conclusion, we have identified, what we believe is a novel regulatory mechanism by which recipient *TIM4* signaling may control metabolic disturbances and the hepatocellular death via an ER stress response in IRI-OLT. As a checkpoint regulator of IR stress and sterile inflammation, hepatic *TIM4* expression may serve as a biomarker in the acute OLT phase, guiding early postoperative management and decision-making for the therapeutic intervention in liver transplant recipients.

## Data Availability

The original contributions presented in the study are included in the article/**Supplementary Material**, further inquiries can be directed to the corresponding author.
