# Supplementary material for: Recipient TIM4 signaling regulates ischemia reperfusion-induced ER stress and metabolic responses in liver transplantation: from mouse-to-human
Source: Front Transplant. 2023 May 19;2:1176384. doi: 10.3389/frtra.2023.1176384 (PMC11235257; doi:10.3389/frtra.2023.1176384)
Supplement: Supplementary file 1 [file Datasheet1.docx]

Recipient TIM4 Signaling Regulates Ischemia Reperfusion-induced ER Stress and Metabolic Responses in Liver Transplantation: From Mouse-to-Human

Hirofumi Hirao^1^, Shoichi Kageyama^2^, Kojiro Nakamura^2^, Kentaro Kadono^1^, Hidenobu Kojima^1^, Yao Siyuan^1^, Douglas G. Farmer^1^, Fady M. Kaldas^1^, Kenneth J. Dery^1*^ and Jerzy W. Kupiec-Weglinski^1^

^1^Dumont-UCLA Transplantation Center, Department of Surgery, Division of Liver and Pancreas Transplantation, Los Angeles, CA, USA

^2^Department of Surgery, Hepato-Biliary-Pancreatic Surgery and Transplantation, Kyoto University, Kyoto, Japan

**Supplemental materials**

**Table of contents**

**Supplemental table 1……………………………………………………………………………..2**

**Supplemental table 2……………………………………………………………………………..3**

**Supplemental table 3……………………………………………………………………………..4**

**Supplemental table 4……………………………………………………………………………..5**

**Supplemental table 5……………………………………………………………………………..6**

**Supplemental table 6……………………………………………………………………………..7**

**Supplemental figure 1……………………………………………………………………………8**

**Supplemental figure 2……………………………………………………………………………9**

**Supplemental figure 3……………………………………………………………………………10**

**Figure S1**

**
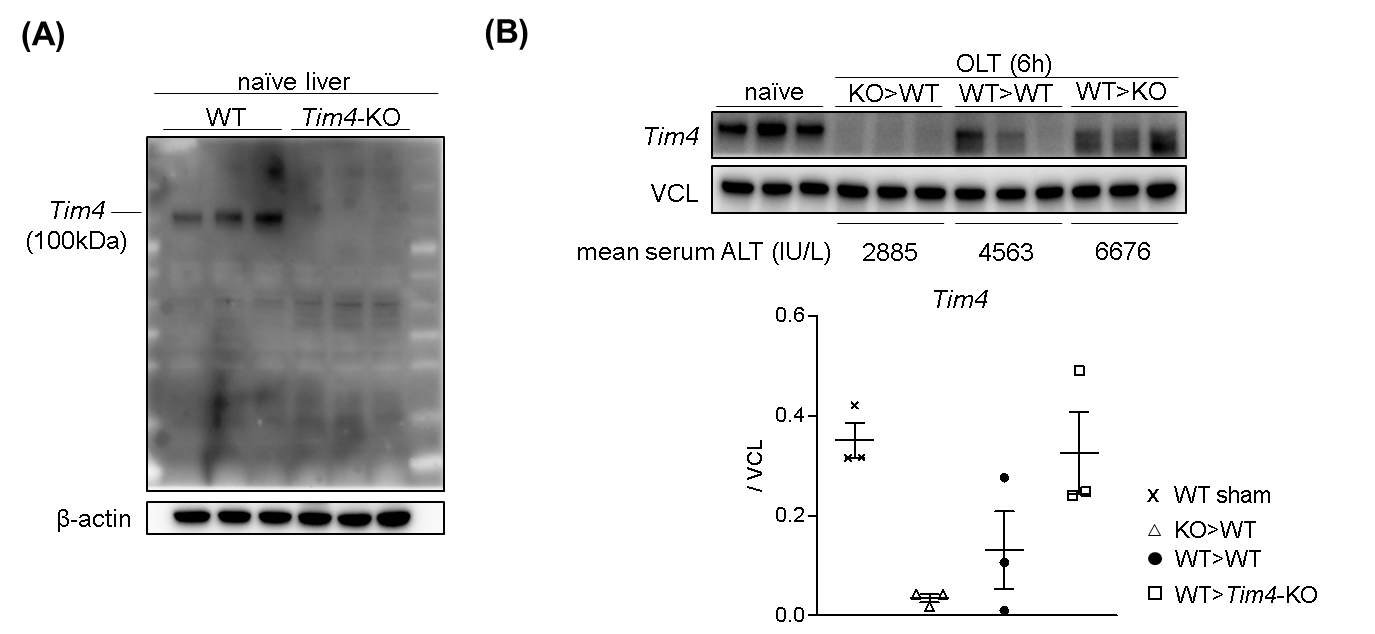
**

**Figure S1: Hepatic *Tim4* expression post-OLT positively correlates with the hepatocellular damage in murine OLT:** Western blot-assisted **(A)** validation of *Tim4* antibody in WT and *Tim4*-KO naïve liver; and **(B)** detection of *Tim4* in OLT (*Tim4*-KO>WT, WT>WT, WT>*Tim4*-KO). β-actin was used as an internal control. Data shown as mean±SEM.

**Figure S2**

**
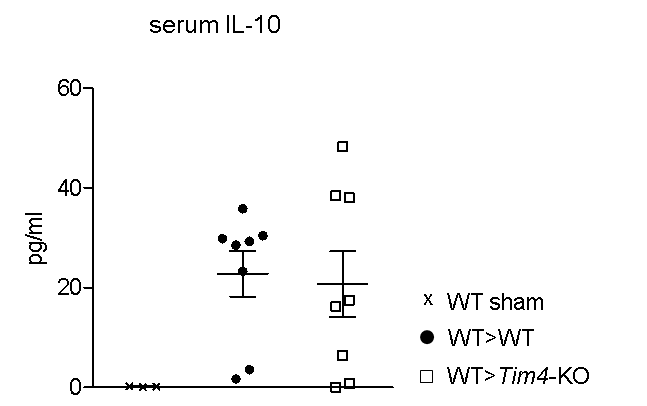
**

**Figure S2: Serum IL-10 levels in OLT mouse recipients:** Serum IL-10 levels (pg/ml) 6h after OLT were measured by ELISA (n=3-8/group). Data shown as mean±SEM.

**Figure S3**

**
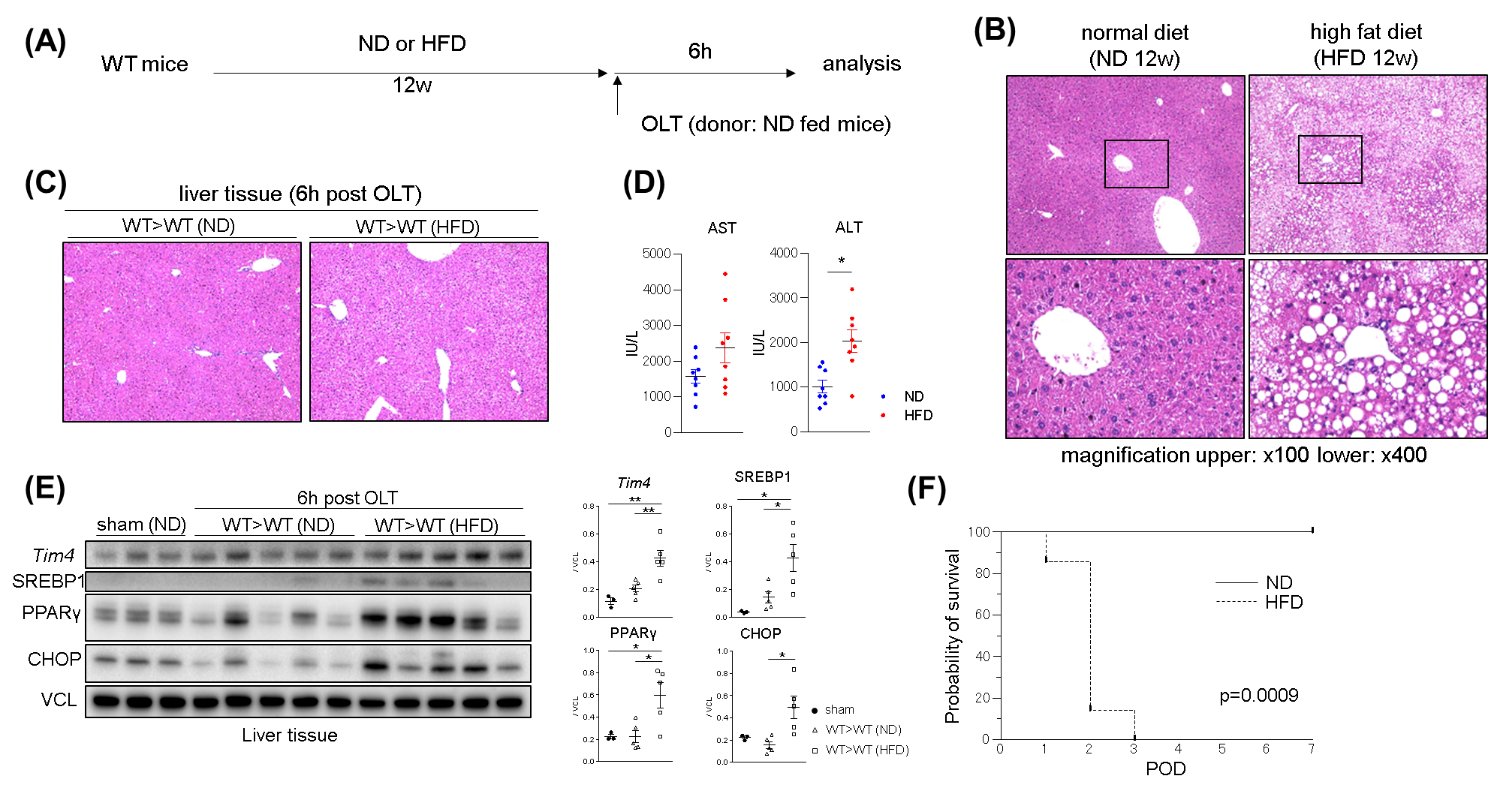
**

**Figure S3:** **OLT in** **HFD-fed mouse recipients: (A)** WT (C57BL/6) mice fed with normal diet (ND) or high fat diet (HFD) for 12 weeks were used as OLT recipients. The donor livers from WT mice fed with ND (12 weeks) were used. **(B)** Representative H&E staining of the liver at 12 weeks (original magnification upper panel: x100, lower panel x400). **(C)** Representative H&E staining 6h after OLT (original magnification x100). **(D)** Serum AST and ALT levels (n=8/group). Data shown as mean±SEM. (*p<0.05, Student t-test, n=8/group) **(E)** WB-assisted detection of *Tim4*, SREBP1, PPARγ and CHOP in sham and post-transplant livers. VCL was used as an internal control. Data shown as mean±SEM. (*p<0.05, **p<0.01, 1-way ANOVA followed by Tukey’s HSD test, n=3-5/group) **(F)** The cumulative probability of overall graft survival. The solid line indicates ND-fed recipients, while the dotted line depicts HFD-fed recipients (Kaplan-Meier method, log-rank test, n=5-7/group).

**Supplemental Methods**

To mimic NASH recipients, 4 weeks old male C57BL/6 mice were fed a normal diet (ND) or high-fat diet (HFD) (D19011801, Research Diets, Inc. New Brunswick, NJ, USA) for 12 weeks, and then blood and tissue samples were collected. The ingredients of HFD is listed in Suppl. Table 6. Some of HFD-fed mice were challenged with syngeneic liver grafts (after 90 min of cold storage).

**Supplemental Results**

***Recipient Tim4-null mutation enhances Tim4 expression in IR-stressed OLT***

We evaluated the correlation between post-transplant liver damage and hepatic *Tim4* expression (sham and OLT livers; KO>WT, WT>WT, WT>KO) (Suppl. Figure 1B). As expected, we failed to detect *Tim4* in *Tim4*-null donor OLT (KO>WT) as compared with WT>WT, while WT livers implanted in *Tim4*-KO recipients exhibited higher *Tim4* expression as compared with the WT>WT group. These results indicate that post-transplant *Tim4* expression can dictate OLT injury.

***HFD-fed recipients show similar phenotype with Tim4 deficient recipients in hepatic protein expression patterns and OLT outcomes***

Since previous report documented *Tim4*+macrophages in AT declined with the progression of NASH (13), we hypothesized HFD-fed mice might have some in common with *Tim4*-null recipients. WT mice were fed with normal diet or high fat diet (HFD, Suppl. Table 6) for 12 weeks and used as OLT recipients to mimic the NASH patients (Suppl. Figure 3A). The development of NASH was confirmed by the histological assessment, with HFD-fed mice showing severe lipid accumulation in the liver (Suppl. Figure 3B). Interestingly, consistent with *Tim4*-null recipients, HFD-fed WT mice exhibited exacerbated hepatocellular necrosis (Suppl. Figure 3C), increased serum transaminase release (Suppl. Figure 3D), and augmented hepatic *Tim4*, SREBP1, PPARγ and CHOP expression (Suppl. Figure 3E). Moreover, all HFD-fed recipients died within 3 days after OLT despite shorter cold storage time (90 min).
